# Comparing outcomes of ILD patients managed in specialised versus non-specialised centres

**DOI:** 10.1186/s12931-022-02143-1

**Published:** 2022-08-27

**Authors:** Pavo Marijic, Larissa Schwarzkopf, Werner Maier, Franziska Trudzinski, Michael Kreuter, Lars Schwettmann

**Affiliations:** 1 Institute of Health Economics and Health Care Management, Helmholtz Zentrum München - German Research Centre for Environmental Health (GmbH), Neuherberg, Germany; 2 Pettenkofer School of Public Health, Munich, Germany; 3 Institute for Medical Information Processing, Biometry and Epidemiology, IBE, LMU Munich, Munich, Germany; 4 Comprehensive Pneumology Centre Munich (CPC-M), Member of the German Centre for Lung Research (DZL), Munich, Germany; 5 IFT-Institut Fuer Therapieforschung, Munich, Germany; 6 Centre for Interstitial and Rare Lung Diseases, Pneumology and Respiratory Critical Care Medicine, Thoraxklinik, University of Heidelberg, German Centre for Lung Research (DZL), Röntgenstr. 1, 69126 Heidelberg, Germany; 7 Department of Economics, Martin Luther University Halle-Wittenberg, Halle, Germany

**Keywords:** Survival, Tertiary care centre, Expert hospitals, Health care costs, Administrative data, Statutory health insurance

## Abstract

**Background:**

Early appropriate diagnosis and treatment of interstitial lung diseases (ILD) is crucial to slow disease progression and improve survival. Yet it is unknown whether initial management in an expert centre is associated with improved outcomes. Therefore, we assessed mortality, hospitalisations and health care costs of ILD patients initially diagnosed and managed in specialised ILD centres versus non-specialised centres and explored differences in pharmaceutical treatment patterns.

**Methods:**

An epidemiological claims data analysis was performed, including patients with different ILD subtypes in Germany between 2013 and 2018. Classification of specialised centres was based on the number of ILD patients managed and procedures performed, as defined by the European Network on Rare Lung Diseases. Inverse probability of treatment weighting was used to adjust for covariates. Mortality and hospitalisations were examined via weighted Cox models, cost differences by weighted gamma regression models and differences in treatment patterns with weighted logistic regressions.

**Results:**

We compared 2022 patients managed in seven specialised ILD centres with 28,771 patients managed in 1156 non-specialised centres. Specialised ILD centre management was associated with lower mortality (HR: 0.87, 95% CI 0.78; 0.96), lower all-cause hospitalisation (HR: 0.93, 95% CI 0.87; 0.98) and higher respiratory-related costs (€669, 95% CI €219; €1156). Although risk of respiratory-related hospitalisations (HR: 1.00, 95% CI 0.92; 1.10) and overall costs (€− 872, 95% CI €− 75; €1817) did not differ significantly, differences in treatment patterns were observed.

**Conclusion:**

Initial management in specialised ILD centres is associated with improved mortality and lower all-cause hospitalisations, potentially due to more differentiated diagnostic approaches linked with more appropriate ILD subtype-adjusted therapy.

**Supplementary Information:**

The online version contains supplementary material available at 10.1186/s12931-022-02143-1.

## Background

Interstitial lung diseases (ILD) include approximately 200 different rare lung diseases with comparable clinical, physiological, radiological or pathological characteristics, but heterogeneous aetiology, prognosis and treatment [1, 2]. ILD, especially fibrosing forms, impose high burdens on patients, as quality of life is impaired, and both morbidity and mortality are high [3]. In recent years, effective therapies for many forms of ILD have been identified, which slow disease progression and improve survival [4]. Owing to the complexity of diagnosing and treating these rare and mostly chronic diseases, a recent European idiopathic pulmonary fibrosis (IPF) patient charter raised the need for improved diagnosis, treatment access, holistic care, disease awareness and palliative care [5]. Additionally, management of comorbidities was demonstrated to be important as they increase mortality [6–8]. However, the most relevant impact might be early and accurate treatment. In this regard, it might be hypothesised that management in specialised ILD centres improves ILD-specific outcomes and contributes to more comprehensive comorbidity management. Support of this hypothesis comes from Lamas et al., who described that delayed access to tertiary care for IPF patients was associated with increased mortality [9]. A further retrospective analysis suggests improved survival in patients managed in a regional ILD clinic [10]. Similarly, differences with respect to in-hospital mortality may exist between patients with IPF admitted to either an academic or a non-academic hospital [11]. However, multicentre data comparing patients managed in specialised ILD centres with those in non-specialised centres for ILDs other than IPF and analysis of other outcomes beyond survival are lacking.

Therefore, the aim of the present study was to compare all-cause mortality, all-cause and respiratory-related hospitalisations and health care costs in ILD patients initially managed in specialised ILD centres with those managed in non-specialised centres. Furthermore, potential differences in pharmaceutical treatment patterns during the 3 months following initial hospital discharge are explored.

## Methods

### Data set and sample selection

This study analysed claims data provided by the largest German statutory health insurance fund (Allgemeine Ortskrankenkasse—AOK), which insured around one third of the German resident population in 2020 (approximately 27 million people) [12]. Membership is open to everyone regardless of factors such as age, income, comorbidities or professional affiliation [13]. Insured persons have free access to a wide range of services with no (outpatient services) or only low co-payments (€10/day for inhospital care). According to national guidelines for secondary data analysis, ethical approval and consent to participate were  not required [14].

The initial data set included all insured adults with an ICD-10 diagnosis of ILD, including idiopathic interstitial pneumonia (IIP) [J84.1], other fibrosing ILD [J84.0, J84.8, J84.9, D48.1], sarcoidosis [D86.0–D86.9], drug-associated ILD [J70.2–J70.4], pneumoconiosis [J62.0–J62.8, J63.0–J63.8], radiation-associated pneumonitis [J70.1], eosinophilic pneumonia [J82], hypersensitivity pneumonitis [J67.9] and connective tissue-associated ILD (CTD) [J99.1], from 1 January 2013 to 31 December 2018. Information on survival was available until 31 December 2019.

Patient selection was performed according to established algorithms [6, 15, 16]. First, patients with an inpatient diagnosis of ILD or a confirmed outpatient diagnosis (outpatient ICD diagnoses in Germany have to be categorized as: ‘Z’ = condition after, ‘A’ = exclusion diagnosis, ‘V’ = suspected diagnosis, and ‘G’ = confirmed diagnosis) from a pulmonologist, an internal medicine specialist or a rheumatologist (the last for CTD only) were preselected. Only patients with at least one relevant diagnostic procedure (bronchoscopy, computerised tomography of the lungs, pulmonary function testing and assessment of antibodies) were considered further. Next, patients with implausible diagnostic patterns were excluded. Implausible diagnostic patterns referred to (1) patients receiving an exclusion diagnosis of ILD after a confirmed ILD diagnosis, (2) patients with radiation-associated pneumonitis, but without diagnosis of malignancy, (3) patients with CTD, but without diagnosis of autoimmune disease, and (4) patients simultaneously assigned to different ILDs. Additionally, patients not continuously enrolled with AOK and those with a baseline or outcome period of less than 1 year were discarded. As we aimed to compare ILD patients initially diagnosed and managed in either specialised ILD centres or non-specialised centres, patients were disregarded if they received their confirmed diagnosis only in an outpatient setting. Furthermore, patients dying during the initial hospital stay were excluded as their outcome period would start right after hospital discharge. Finally, we excluded patients with missing variables. Missing variables could only occur for patient characteristics such as area of residence or employment, but not for claims.

Specialised ILD centres were identified based on competency requirements of the European Reference Network on Rare Lung Diseases (ERN-Lung) valid until 2018 and including minimum patient numbers as well as procedures for expert centres [17]. More specifically, at least 400 patients treated per year were required, with at least 150 newly managed patients. In addition, conduct of at least 50 bronchoalveolar lavages (BAL), 30 endobronchial ultrasound-guided transbronchial needle aspirations (EBUS-TBNA) and 20 surgical lung biopsies or transbronchial cryobiopsies (SLB-TC) was necessary to be classified as a specialised ILD centre. The procedures were identified via the OPS codes (German version of the International Classification of Procedures in Medicine) presented in Additional file 1: Table S1. We adapted the numbers outlined above to the expected proportion of patients insured by the AOK using the market share of AOK in 2014 as reference. Therefore, 120 ILD patients (including 45 newly managed), 15 BAL, 9 EBUS-TBNA and 6 SLB-TC classified a hospital as a specialised ILD centre. In case a hospital did not fulfil the required criteria in a particular year but in both adjacent years, it was also considered a specialised ILD centre in the ‘gap year’. Hospitals not meeting these criteria were considered non-specialised centres. Following this definition, we included patients treated in specialized ILD-centres in the intervention group, while patients initially treated in a hospital not meeting these conditions belonged to the control group. Patients who were transferred directly from a non-specialised centre to a specialized ILD centre after initial diagnoses were also included in the intervention group, as this was considered an “uninterrupted” hospitalization.

### Outcome variables

Specialised ILD centre- and non-specialised centre-managed patients were compared regarding 2-year all-cause mortality, 1-year all-cause as well as respiratory-related hospitalisation and 1-year overall and respiratory-related health care costs. Outcomes were calculated on a date basis starting at the date of the initial diagnosis, which also represents the date of confirmed diagnosis.

The following primary diagnoses constituted respiratory-related hospitalisations [18]: respiratory infection [A481, B250, J09–J22, J40], pneumothorax [J93], pulmonary embolism [I26], pulmonary hypertension and right heart disease [I50, I270, I272, I278, I279], respiratory insufficiency [J96], other chronic and acute lung diseases [J40–J47] and the ICD-10 codes for the ILD of the respective patients as presented above.

1-year health care costs following the confirmed diagnosis were calculated based on outpatient physician care, inpatient care and pharmaceuticals. Outpatient physician costs refer to all costs in the outpatient sector provided by physicians including all specialties. Inpatient costs comprise all services provided by hospitals, while pharmaceutical costs refer to all medications retrieved by patients from a pharmacy. Respiratory-related outpatient and inpatient costs referred to the respiratory-related ICD codes outlined above. Respiratory-related drug costs referred to filed prescriptions of antifibrotic drugs (pirfenidone, nintedanib), steroids (glucocorticoids, corticosteroids), immunosuppressants, acetylcysteine, sildenafil and drugs for pulmonary arterial hypertension identified by the Anatomical Therapeutic Chemical (ATC) codes presented in Additional file 1: Table S2. As outpatient physician costs were available per quarter of the year, we assigned costs incurred in the quarter of the initial hospital discharge proportionally to days before and after discharge. Within the last observation quarter, costs were proportionally allocated to days under observation. In contrast, inpatient costs were available on a daily base. If a hospital stay exceeded the 1-year observation period, costs were distributed proportionally to in-hospital days within the observation period. Costs for pharmaceuticals were calculated for the day the prescription was filled.

In addition to the main outcomes, differences in pharmaceutical treatment patterns were investigated. Therefore, the onset of ILD-relevant drugs in the 3 months after initial hospital discharge was explored. Here, we considered antifibrotic drugs, immunosuppressants, steroids, anti-clotting drugs, anti-acid drugs, anti-depressants, anti-diabetic drugs, drugs for obstructive airway disease, drugs for pulmonary arterial hypertension, drugs for heart insufficiency/cardiac arrhythmia and drugs for cardiovascular disease (Additional file 1: Table S2).

### Covariates

We used stabilised inverse probability of treatment weighting (IPTW) based on covariate balancing propensity scores (CBPS) [19, 20] to adjust for baseline variables. Based on clinical expertise and pre-existing literature, the following covariates were considered: age in years, sex (male/female), residential area (major city, urban, rural, remote rural) [21], the 2015 version of the German Index of Multiple Deprivation (GIMD 2015) as a proxy for individual socioeconomic background [22, 23], information on nursing home residency (yes/no), care level reflecting need for nursing care, the patient’s employment status (yes/no) and comorbidity burden. Comorbidities were captured based on the Elixhauser Index [24]. To reflect ILD-specific comorbidity more comprehensively, pulmonary hypertension and lung cancer were separated from the corresponding Elixhauser categories [6, 25] and gastro-oesophageal reflux disease, obstructive sleep apnoea syndrome, ischaemic heart disease and thromboembolism were also considered [6]. The Elixhauser score was calculated by summing up all Elixhauser categories without the ILD-specific comorbidities, which were included separately in dummy-coded format. Dummy-coded Elixhauser categories were additionally included in the propensity score model, if they had a prevalence of at least 5% in the treatment group.

Furthermore, we adjusted for medical treatment with ILD-relevant and comorbidity-related pharmaceuticals (Additional file 1: Table S2) in the 3 months prior to the initial hospitalisation. With respect to the utilisation of health care services, the respective time frame was accounted for by including information on all-cause and respiratory-related hospitalisation as well as contacts with all outpatient physicians and a dummy-coded variable for treatment by a pulmonologist. Moreover, we incorporated outpatient, inpatient and pharmaceutical costs in the year prior to the initial hospital admission. Finally, the year of the confirmed diagnosis was accounted for.

To achieve a further reduction in group differences, restricted cubic splines with five knots for the continuous variables were applied [26]. Balance of covariates between the groups was examined via standardised mean differences (SMD) and the Kolgomorov–Smirnov test [19], with SMDs less than 0.1 indicating a good balance [27].

### Statistical analysis

We depicted IPTW-weighted Kaplan–Meier plots for survival and hospitalisations. Cox Proportional Hazard models with sandwich estimators were used to analyse the related group differences as hazard ratios (HR) with 95% confidence intervals (CI). Depending on the share of zero costs, we performed IPTW-weighted one-part and two-part Generalized Linear Gamma models to account for the skewedness of cost data [28]. Euro amounts were calculated based on recycled predictions [29] with 95% CIs obtained from 1000 bootstrap replications.

To contrast pharmaceutical treatment patterns in the 3 months after discharge exploratively, we applied weighted logistic regression models.

Furthermore, we performed three subgroup analyses addressing patients with IIP, sarcoidosis and all other ILD. A new propensity score model was calculated for each subgroup, although we used the same statistical methods as described above.

An additional sensitivity analysis considered that patients initially managed in non-specialised centres might have received treatment in specialised ILD centres in the year after the confirmed diagnosis. Patients who were also managed in specialised ILD centres were censored for the time-to-event analyses at the day they were admitted to the specialised ILD centre and excluded for the cost analyses.

Results were considered significant if the 95% CIs of the differences did not contain ‘1’ in the Cox models and ‘0’ in the cost analyses. All analyses were conducted in R Software version 4.0.3 and SAS version 9.4.

## Results

### Population characteristics

**Fig. 1:**
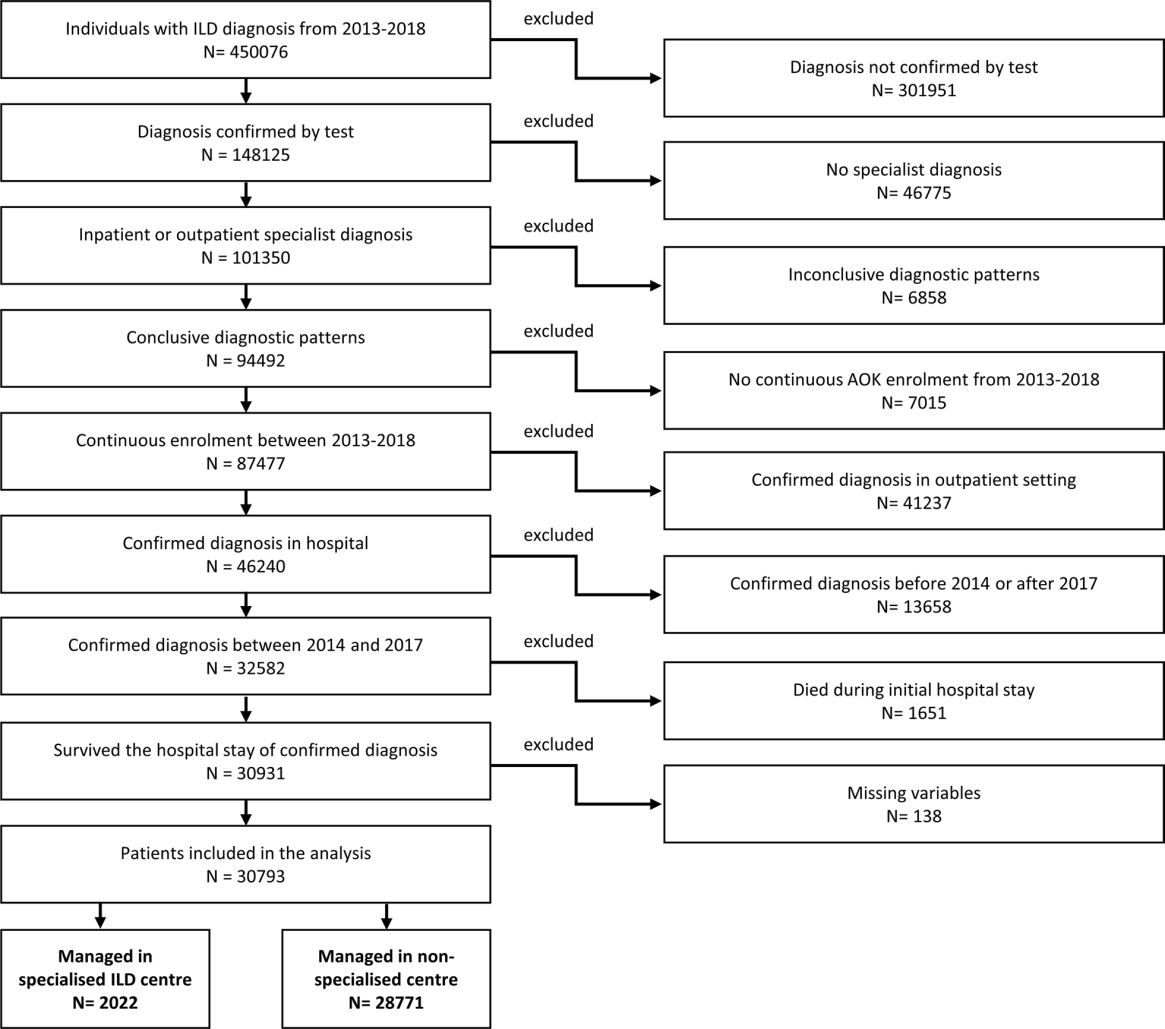
Participant flow of the study population

Of the 30,793 patients who met the inclusion criteria, 2022 (6.6%) were initially managed in one of the seven specialised ILD centres classified according to the ERN-Lung requirements. The remainder were managed in 1156 non-specialised centres (Fig. 1). Specialised ILD centres treated on average 154 ILD patients per year, whereas non-specialised centres treated about 10 ILD patients. Additional file 2: Table S3 provides the numbers of procedures per clinic type. Patients managed in specialised ILD centres were younger, less often diagnosed with IIPs and more often employed (Table 1). They lived in less deprived areas and had fewer comorbid conditions. After IPTW weighting, the groups were very well balanced with all SMDs at 0.001 or lower and no difference in the distribution of the continuous variables (Additional file 2: Figure S1). The subgroups are characterised in Additional file 2: Tables S4–S6.

**Table 1:** Patient characteristics with raw and IPTW-weighted standardised mean differences

	Unweighted			IPTW weighted
	Non-specialised centre (N = 28,771)	Specialised ILD centre (N = 2022)	SMD	SMD
Age (years), mean (SD)	67.4 (15.0)	64.0 (14.1)	0.232	< 0.001
Sex (female), n (%)	12,306 (42.8)	779 (38.5)	0.087	< 0.001
ILD entity [ICD-10], n (%)			0.241	< 0.001
Idiopathic interstitial pneumonia [J84.1]	11,839 (41.1)	716 (35.4)		
Other fibrosing ILDs [J84.0, J84.8, J84.9, D48.1]	6147 (21.4)	507 (25.1)		
Sarcoidosis [D86.0–D86.9]	6049 (21.0)	448 (22.2)		
Drug-associated ILDs [J70.2–J70.4]	488 (1.7)	37 (1.8)		
Pneumoconiosis [J62.0–J62.8, J63.0–J63.8]	826 (2.9)	38 (1.9)		
Radiation-associated pneumonitis [J70.1]	438 (1.5)	63 (3.1)		
Eosinophilic pneumonia [J82]	624 (2.2)	54 (2.7)		
Hypersensitivity pneumonitis [J67.9]	773 (2.7)	97 (4.8)		
Connective tissue-associated ILD [J99.1]	1587 (5.5)	62 (3.1)		
GIMD 2015, n (%)			0.603	0.001
Q1 (least deprived quintile)	4656 (16.2)	650 (32.1)		
Q2	6131 (21.3)	336 (16.6)		
Q3	5678 (19.7)	80 (4.0)		
Q4	6121 (21.3)	507 (25.1)		
Q5 (most deprived quintile)	6185 (21.5)	449 (22.2)		
Residential area, n (%)			0.309	0.001
Major city	6516 (22.6)	562 (27.8)		
Urban districts	10,485 (36.4)	786 (38.9)		
Rural districts	6023 (20.9)	485 (24.0)		
Remote rural districts	5747 (20.0)	189 (9.3)		
Nursing home residency, n (%)	975 (3.4)	25 (1.2)	0.144	< 0.001
Care dependency, n (%)			0.282	< 0.001
No care level	21,954 (76.3)	1758 (86.9)		
Care level 1	3253 (11.3)	112 (5.5)		
Care level 2	2449 (8.5)	98 (4.8)		
Care level 3	823 (2.9)	42 (2.1)		
Care level 4	232 (0.8)	9 (0.4)		
Care level 5	60 (0.2)	3 (0.1)		
Employment, n (%)	5156 (17.9)	527 (26.1)	0.198	< 0.001
Comorbidities Elixhauser score, mean (SD)	3.40 (2.42)	3.05 (2.30)	0.149	< 0.001
Comorbidities modified Elixhauser categories, n (%)				
Congestive heart failure	5920 (20.6)	305 (15.1)	0.144	< 0.001
Cardiac arrhythmias	6278 (21.8)	334 (16.5)	0.135	< 0.001
Valvular disease	3207 (11.1)	186 (9.2)	0.064	< 0.001
Peripheral vascular disorders	5401 (18.8)	309 (15.3)	0.093	< 0.001
Hypertension, uncomplicated	14,881 (51.7)	968 (47.9)	0.077	< 0.001
Hypertension, complicated	3138 (10.9)	200 (9.9)	0.033	< 0.001
Chronic pulmonary disease	10,261 (35.7)	745 (36.8)	0.025	< 0.001
Diabetes, uncomplicated	3849 (13.4)	229 (11.3)	0.062	< 0.001
Diabetes, complicated	5091 (17.7)	323 (16.0)	0.046	< 0.001
Hypothyroidism	2956 (10.3)	218 (10.8)	0.017	< 0.001
Renal failure	4641 (16.1)	241 (11.9)	0.122	< 0.001
Liver disease	3696 (12.8)	284 (14.0)	0.035	< 0.001
Solid tumour without metastasis	2999 (10.4)	199 (9.8)	0.019	< 0.001
Rheumatoid arthritis/collagen vascular diseases	3604 (12.5)	201 (9.9)	0.082	< 0.001
Obesity	4900 (17.0)	324 (16.0)	0.027	< 0.001
Depression	6190 (21.5)	436 (21.6)	0.001	< 0.001
Comorbidities IPF specific, n (%)				
Coronary heart disease	7885 (27.4)	458 (22.7)	0.110	< 0.001
Gastro-oesophageal reflux disease	3939 (13.7)	293 (14.5)	0.023	< 0.001
Obstructive sleep apnoea syndrome	1527 (5.3)	140 (6.9)	0.067	< 0.001
Thrombosis	852 (3.0)	68 (3.4)	0.023	< 0.001
Lung cancer	1016 (3.5)	146 (7.2)	0.164	< 0.001
Pulmonary hypertension	844 (2.9)	64 (3.2)	0.013	< 0.001
Drug treatments, n (%)				
Immunosuppressants	672 (2.3)	39 (1.9)	0.028	< 0.001
Acetylcysteine	803 (2.8)	79 (3.9)	0.062	< 0.001
Glucocorticoids, corticosteroids	6107 (21.2)	417 (20.6)	0.015	< 0.001
Treatment with anti-clotting drugs	5850 (20.3)	322 (15.9)	0.115	< 0.001
Treatment with anti-acid drugs	11,101 (38.6)	695 (34.4)	0.088	< 0.001
Treatment with anti-depressants	3620 (12.6)	192 (9.5)	0.099	< 0.001
Treatment with anti-diabetic drugs	4877 (17.0)	298 (14.7)	0.061	< 0.001
Treatment with drugs against obstructive airway disease	6104 (21.2)	474 (23.4)	0.053	< 0.001
Treatment of pulmonary hypertension	191 (0.7)	15 (0.7)	0.009	< 0.001
Treatment of heart insufficiency/cardiac arrhythmia	9019 (31.3)	449 (22.2)	0.208	< 0.001
Treatment of cardiovascular disease	16,218 (56.4)	995 (49.2)	0.144	< 0.001
Hospitalisations in 3 months before treatment, n (%)				
All cause	10,332 (35.9)	847 (41.9)	0.123	< 0.001
Respiratory related	3039 (10.6)	260 (12.9)	0.071	< 0.001
Use of outpatient services in the year before treatment				
Contacts with physicians overall, mean (SD)	11.8 (8.9)	12.8 (8.2)	0.117	< 0.001
Contacts with pulmonologists, n (%)	5673 (19.7)	830 (41.0)	0.477	< 0.001
Costs in the year before diagnosis in €, mean (SD)				
Outpatient costs	1340 (2605)	1288 (1857)	0.023	< 0.001
Inpatient costs	10,080 (14,917)	10,137 (17,512)	0.004	< 0.001
Pharmaceutical costs	2644 (8367)	2315 (7113)	0.042	< 0.001
Year of confirmed diagnosis, mean (SD)			0.130	< 0.001
2014	7333 (25.5)	467 (23.1)		
2015	7182 (25.0)	457 (22.6)		
2016	7237 (25.2)	490 (24.2)		
2017	7019 (24.4)	608 (30.1)		

GIMD 2015: German Index of Multiple Deprivation, year 2015; IPTW: Inverse probability of treatment weighting; Q: Quintile; SD: Standard deviation; SMD: Standardised mean difference

### Mortality

**Fig. 2:**
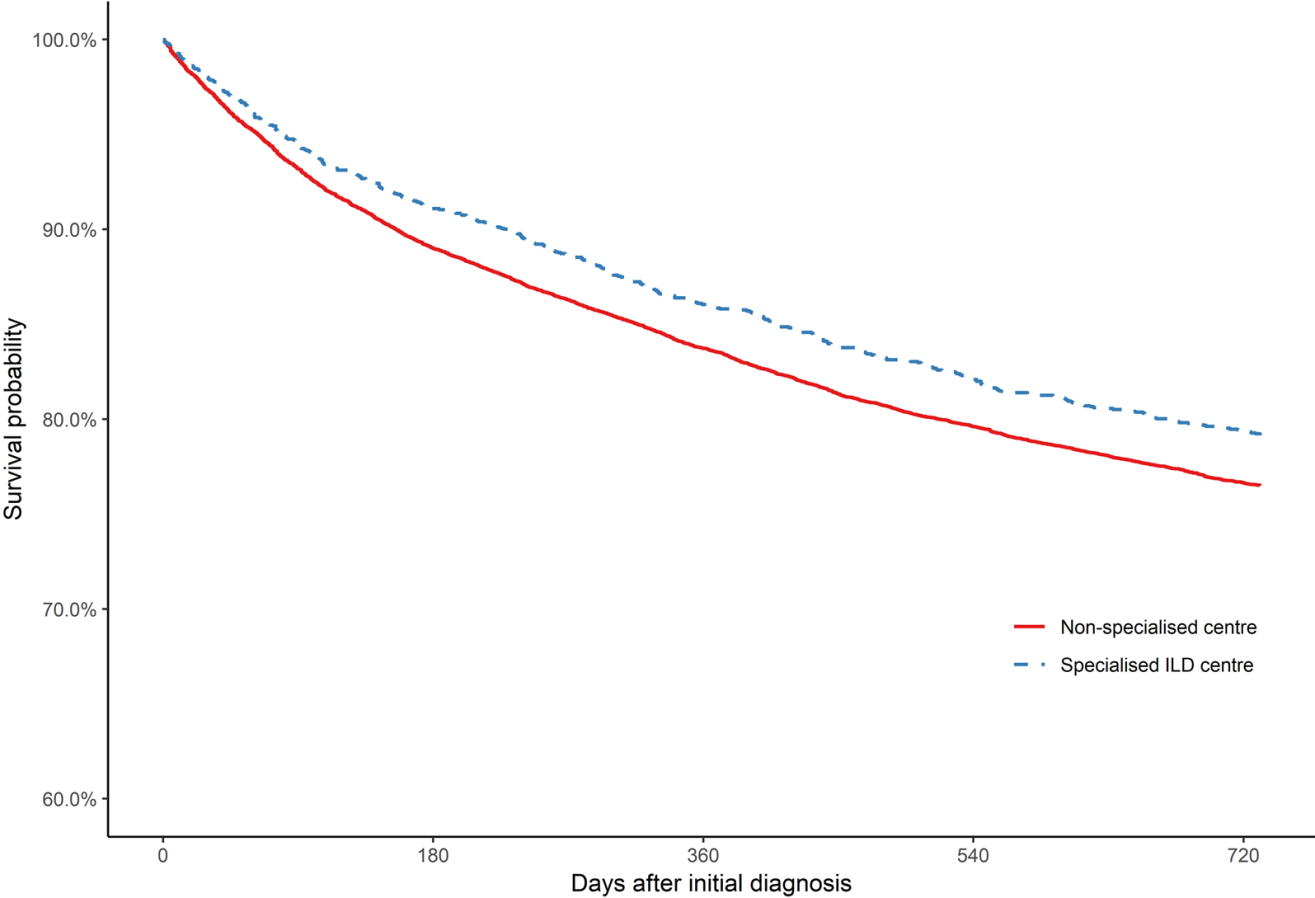
IPTW-weighted Kaplan–Meier plots for 2-year all-cause mortality

In all, 29% of patients managed in non-specialised centres, but only 21% of patients managed in specialised ILD centres died during the 2-year follow-up. The mean follow-up time was 594 days for patients managed in non-specialised centres and 635 days for patients managed in specialised ILD centres. Hence, the 2-year survival probability for patients initially managed in specialised ILD centres was improved (Fig. 2). Accordingly, there was a significant survival benefit (Table 2). The positive association observed between specialised ILD centre management and mortality was most pronounced for IIPs (Table 2). However, the effect sizes obtained for patients with sarcoidosis and other ILDs were similar to the main analysis. The unweighted estimates are presented in Additional file 3: Table S7.

**Table 2:** IPTW-weighted Cox Proportional Hazard models for 2-year mortality and 1-year all-cause and respiratory-related hospitalisation

	All patients	Subgroup analyses		
		Idiopathic interstitial pneumonia	Sarcoidosis	Other interstitial lung diseases
	HR (95% CI)	HR (95% CI)	HR (95% CI)	HR (95% CI)
2-year all-cause mortality				
Specialised ILD centre vs. non-specialised centre	0.87 (0.78, 0.96)*	0.81 (0.70, 0.94)*	0.89 (0.59, 1.34)	0.90 (0.78, 1.05)
1-year all-cause hospitalisation				
Specialised ILD centre vs. non-specialised centre	0.93 (0.87, 0.98)*	0.90 (0.82, 1.00)	0.94 (0.80, 1.10)	0.94 (0.86, 1.02)
1-year respiratory-related hospitalisation				
Specialised ILD centre vs. non-specialised centre	1.00 (0.92, 1.10)	0.98 (0.86, 1.12)	0.89 (0.70, 1.13)	1.03 (0.90, 1.19)

CI: Confidence interval, HR: Hazard ratio, IPTW: Inverse probability of treatment weighting

^*^Statistically significant results

### Hospitalisation

**Fig. 3:**
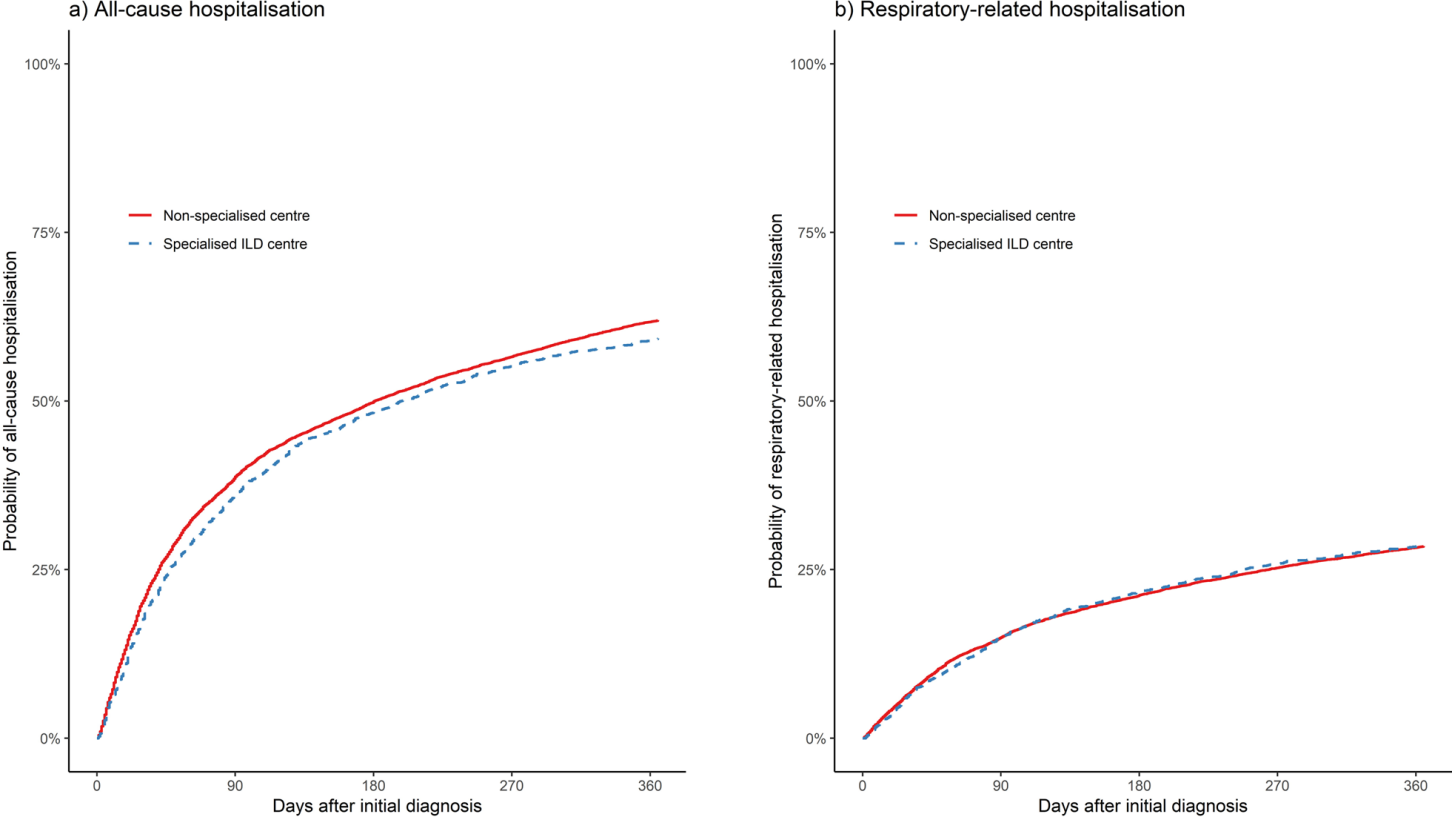
IPTW-weighted cumulative incidence curves for 1-year all-cause and respiratory-related hospitalisation. Cumulative incidence curves (1 minus the Kaplan–Meier risk) depict the period from the confirmed diagnosis

During the 1-year follow-up, 64% of patients managed in non-specialised centres and 58% of patients managed in specialised ILD centres were hospitalised. In both groups, 29% and 27%, respectively, were hospitalised for respiratory-related reasons. Hence, there were fewer all-cause hospitalisations in specialised ILD centre-managed patients (Fig. 3a), although the pattern for respiratory-related hospitalisations was rather similar (Fig. 3b). The hazard for all-cause hospitalisation was significantly reduced in specialised ILD centre-managed patients, whereas there was no difference regarding respiratory-related hospitalisations (Table 2). In the subgroup analyses, similar estimates below the significance threshold were observed for all-cause hospitalisations. In sarcoidosis patients, a lower, non-significant effect was demonstrated regarding respiratory-related hospitalisation. The unweighted estimates are presented in Additional file 3: Table S7.

### Costs

Overall costs were similar between both groups but, when focusing on the distinct cost categories, we observed lower inpatient and higher pharmaceutical costs for patients managed in specialised ILD centres (Table 3). Respiratory-related costs were higher in specialised ILD centre-managed patients, as higher respiratory-related pharmaceutical costs overcompensated for lower respiratory-related inpatient costs. In the subgroup analyses, costs for patients with IIP were similar to those in the main analysis (Additional file 3: Table S8). For patients with sarcoidosis and other ILD, overall costs for specialised ILD centre-managed patients were significantly lower on account of reduced inpatient costs (Additional file 3: Tables S9, S10).

**Table 3:** IPTW-weighted model estimated expenditures of 1-year costs after confirmed diagnosis and related cost differences with 95% confidence intervals

	Non-specialised centre	Specialised ILD centre	Difference (in €)
	Costs in € (95% CI)	Costs in € (95% CI)	
Overall			
Total^a^	13,953 (13,554; 14,353)	13,082 (12,252; 13,906)	− 872 (− 75; 1817)
Inpatient^b^	8814 (8482; 9164)	6970 (6364; 7606)	− 1845 (− 2609; − 1133)*
Outpatient^a^	1378 (1349; 1406)	1363 (1266; 1453)	− 15 (− 117; 76)
Pharmaceuticals^a^	3761 (3590; 3927)	4753 (4287; 5243)	992 (488; 1562)*
Respiratory-related			
Total^b^	3717 (3546; 3897)	4385 (3966; 4855)	669 (219; 1156)*
Inpatient^b^	2206 (2076; 2352)	1762 (1543; 1993)	− 444 (− 709; − 175)*
Outpatient^b^	482 (467; 496)	498 (452; 552)	17 (− 32; 70)
Pharmaceuticals^b^	1029 (936; 1130)	2121 (1754; 2497)	1092 (759; 1464)*

Estimation based on weighted one- and two-part generalized linear gamma models via recycled predictions approach with 1000 bootstrap replicates

CI: Confidence interval, ILD: Interstitial lung disease, IPTW: Inverse probability of treatment weighting

^*^Statistically significant results

^a^One-part model

^b^Two-part model

### Explorative analysis of pharmaceutical treatment patterns

Patients managed in specialised ILD centres more often received prescriptions of antifibrotics, immunosuppressants and drugs for pulmonary hypertension in the 3 months after initial discharge (Table 4). Follow-up treatment with anti-clotting drugs, medication against obstructive airway disease and medication for heart insufficiency/cardiac arrhythmia was less frequent for patients managed in specialised ILD centres. The crude percentages for the periods before and after the initial diagnosis are presented in Additional file 4: Table S11.

**Table 4:** IPTW-weighted logistic regression for change in pharmaceutical treatment patterns in the 3 months after confirmed diagnosis

	OR (95% CI)
Antifibrotics	2.43 (1.92, 3.03)*
Immunosuppressants	1.28 (1.01, 1.60)*
Acetylcysteine	1.21 (0.97, 1.48)
Glucocorticoids, corticosteroids	1.03 (0.97, 1.08)
Treatment with anti-clotting drugs	0.87 (0.79, 0.96)*
Treatment with anti-acid drugs	0.97 (0.93, 1.02)
Treatment with anti-depressants	0.92 (0.80, 1.05)
Treatment with anti-diabetic drugs	1.00 (0.90, 1.11)
Treatment with drugs against obstructive airway disease	0.86 (0.78, 0.93)*
Treatment of pulmonary hypertension	1.74 (1.24, 2.37)*
Treatment of heart insufficiency/cardiac arrhythmia	0.86 (0.80, 0.93)*
Treatment of cardiovascular disease	0.98 (0.94, 1.03)

IPTW-weighted logistic regression for probability of receiving the investigated pharmaceutical in the 3 months after the confirmed diagnosis. Propensity score model included information on drug prescriptions before the diagnosis

CI: Confidence interval, IPTW: Inverse probability of treatment weighting, OR: Odds ratio

^*^Statistically significant results

### Sensitivity analyses

Of the 28,771 patients initially managed in non-specialised centres, 450 (1.6%) received follow-up treatment in a specialised ILD centre within 12 months. Censoring/exclusion of these patients hardly affected the results obtained in the main analysis (Additional file 5: Table S12).

## Discussion

We have reported that ILD patients initially managed in specialised ILD centres have improved mortality and fewer all-cause hospitalisations than ILD patients managed in non-specialised centres.

Our results are in line with a study by Lamas et al. showing that delayed referral to tertiary care centres is associated with an increased mortality rate in IPF [9]. However, their study included only patients with established IPF, whereas we considered newly diagnosed and managed patients with various ILDs, i.e. diagnosis and initial treatment in specialised ILD centres vs. non-specialised centres. Thus, the positive association with survival and all-cause hospitalisation seen for specialised ILD centre care may result from a different management approach compared with non-specialised centres. In line with this, we noticed that pharmaceutical treatment patterns more frequently included antifibrotic and immunosuppressant drugs as well as medical treatment for pulmonary hypertension in the 3 months following a confirmed diagnosis. This finding suggests more subtype-tailored and perhaps more holistic management initiated by specialised centres. Our results support recent observations using registry data from ILD centres illustrating improved survival in IPF patients with antifibrotic treatment [30]. Noteworthy, in our study sample, the number of patients with fibrotic ILDs whose antifibrotic treatment was initiated early is low, which might be attributable to the time analyzed (2013–2018) and reflect comparable registry data in Germany from a similar time point [31]. We assume that meanwhile—in light of further evidence [30, 32, 33] and current guideline recommendations [34]—early antifibrotic treatment for IPF and progressive pulmonary fibrosis has been substantially increasing. This might be associated with lower rates of acute exacerbations and subsequent respiratory-related hospitalizations as supported by recent analyses [25].

The most pronounced positive effect on all-cause hospitalisation and mortality reported in our study was observed for patients with IIPs. Thus, our data support the patients’ demand for early and holistic management [35]. The reduced mortality effects noticed might also be associated with fewer ILD-related complications. In this regard, pulmonary hypertension is a frequent complication in ILD patients that is associated with increased mortality [4, 36]. Although no approved therapy for ILD-associated pulmonary hypertension yet exists in Europe, expert consensus recommends management of the patients concerned in expert centres. Furthermore, inhaled treprostinil recently achieved positive effects on outcomes [37]. A recent international survey could not reveal significant management differences regarding acute exacerbations—an often lethal complication of fibrosing ILDs—between specialised ILD centres and non-specialised centres [38]. This might explain why our data do not suggest that management at specialised ILD centres has an impact on patterns of respiratory-related hospitalisation. Nevertheless, respiratory-related hospitalisation costs were lower for patients managed at specialised ILD centres, which suggests shorter utilisation of respiratory-related inpatient services. Although no relevant differences in respiratory-related hospitalisations were found, the benefits regarding all-cause hospitalisations were striking. Reasons for this effect might be related to improved management of comorbidities and the effects of ILD-specific therapy on comorbidities that significantly affect outcomes in ILDs [6, 18].

From an economic perspective, treatment at a specialised ILD centre was not associated with higher overall costs in the year after the confirmed diagnosis, even though patients were more likely to receive more expensive drugs. In fact, higher pharmaceutical costs were offset by lower inpatient costs.

The sensitivity analysis revealed that only a small proportion (1.6%) of patients initially managed in non-specialised centres received follow-up treatment in specialised ILD centres in the following year.

On the one hand, our study has limitations. First, there might be some misclassification of ILD patients, although we applied established selection criteria. Second, despite the comprehensive IPTW weighting, residual confounding might remain. Here, especially the missing information on clinical data such as forced vital capacity (FVC) or ILD-status (acute vs. chronic) at admission might have had an impact. This is of particular importance as many specialized ILD centres have respiratory intensive care units, which in comparison to general wards have been demonstrated to be associated with a survival benefit in acute respiratory patients [39]. The provision of a specialised unit for acute respiratory patients may facilitate recovery in several critical patients including those with ILD.

Third, the algorithm to identify expert centres is debatable. However, by using the definition from the ERN-Lung, we believe that the most relevant ILD centres were identified. Fourth, by analysing the pharmaceutical treatment patterns, we used general prescription information, but we are not able to link this directly to the hospital. However, it is generally understood that the change in treatment regime results mainly from the initial ILD hospitalisation.

On the other hand, the study provides novel insights into the impact of disease management by expert hospitals in patients with ILD considering several ILD subtypes in a large sample. Furthermore, our data set provides real world information on patients and thereby is less prone to selection bias. This allowed us to identify real world treatment patterns and health care utilisation of the patients included. Additionally, with our study design, we focused on patients who were eligible for treatment in both types of hospitals, thus reducing the bias associated with typically more severely ill patients treated in specialised hospitals.

## Conclusion

Our study suggests that patients with ILD initially managed in specialised ILD centres have significantly improved survival and a lower probability of 1-year all-cause hospitalisations. It supports the hypothesis that patients in specialised ILD centres might be managed in a more tailored fashion compared with non-specialised centres. Improved cooperation between specialised ILD centres and non-specialised centres might be beneficial for ILD patients.

## Supplementary Information

**Additional file 1: Table S1.** OPS-codes for identification of procedures relevant for ILD centre classification. **Table S2.** Drug-related Anatomical Therapeutic Chemical codes.

**Additional file 2: Table S3.** Number of patients treated, patients newly treated, and procedures to classify specialised ILD centres. **Table S4.** Characteristics of patients with idiopathic interstitial pneumonia with raw and IPTW-weighted standardized mean differences. **Table S5.** Characteristics of patients with sarcoidosis with raw and IPTW-weighted standardized mean differences. **Table S6.** Characteristics of patients with other interstitial lung diseases with raw and IPTW-weighted standardized mean differences. **Figure S1.** Balancing of covariates with standardized absolute mean differences and Kolgomorov–Smirnov test.

**Additional file 3: Table S7.** Estimates of unweighted Cox Proportional Hazard Models for 2-year mortality and 1-year hospitalisation after confirmed diagnosis for main analysis and subgroups. **Table S8.** IPTW-weighted model estimated expenditures of 1-year costs and related cost differences for patients with idiopathic interstitial pneumonia. **Table S9.** IPTW-weighted model estimated expenditures of 1-year costs and related cost differences for patients with sarcoidosis. **Table S10.** IPTW-weighted model estimated expenditures of 1-year costs and related cost differences for patients with other interstitial lung diseases.

**Additional file 4: Table S11.** Explorative analysis for change in pharmaceutical treatment patterns in the 3 month after confirmed diagnosis.

**Additional file 5: Table S12.** Sensitivity analyses with unweighted and IPTW-weighted Cox Proportional Hazard models for 2-year mortality and 1-year all-cause and respiratory-related hospitalisation.

## Data Availability

The authors confirm that the data utilized in this study cannot be made available in the manuscript, the additional files, or in a public repository due to German data protection laws (‘Bundesdatenschutzgesetz’, BDSG). Therefore, they are stored on a secure drive in the AOK Research Institute (WIdO) to facilitate replication of the results. Generally, access to data of statutory health insurance funds for research purposes is possible only under the conditions defined in German Social Law (SGB V § 287). Requests for data access can be sent as a formal proposal specifying the recipient and purpose of the data transfer to the appropriate data protection agency. Access to the data used in this study can only be provided to external parties under the conditions of the cooperation contract of this research project and after written approval by the health insurance. For assistance in obtaining access to the data, please contact wido@wido.bv.aok.de.

## Abbreviations

AOK: Allgemeine Ortskrankenkasse
ATC: Anatomical Therapeutic Chemical
BAL: Bronchoalveolar lavages
CBPS: Covariate balancing propensity scores
CI: Confidence interval
CTD: Connective tissue-associated interstitial lung disease
EBUS-TBNA: Endobronchial ultrasound-guided transbronchial needle aspirations
ERN-Lung: European Reference Network on Rare Lung Diseases
FVC: Forced vital capacity
GIMD: German Index of Multiple Deprivation
HR: Hazard ratio
IIP: Idiopathic interstitial pneumonia
ILD: Interstitial lung disease
IPF: Idiopathic pulmonary fibrosis
IPTW: Inverse probability of treatment weighting
SLB-TC: Surgical lung biopsies or transbronchial cryobiopsies
SMD: Standardized mean difference
