# Supplementary material for: Comparing outcomes of ILD patients managed in specialised versus non-specialised centres
Source: Respir Res. 2022 Aug 27;23:220. doi: 10.1186/s12931-022-02143-1 (PMC9420269; doi:10.1186/s12931-022-02143-1)
Supplement: Supplementary file 1 — Additional file 1: Table S1. OPS-codes for identification of procedures relevant for ILD centre classification. Table S2. Drug-related Anatomical Therapeutic Chemical codes. [file 12931_2022_2143_MOESM1_ESM.docx]

Additional file 1: Table S1 OPS-codes for identification of procedures relevant for ILD centre classification

| **Procedures** | **OPS-codes** |
| --- | --- |
| Bronchoalveolar lavage (BAL) | 1-160.01 |
| Endobronchial ultrasound-guided transbronchial needle aspirations (EBUS-TBNA) | 1-430.2; 1-691.0 |
| Surgical lung biopsies or transbronchial cryobiopsies | 1-426.3 |

OPS: German Version of the International Classification of Procedures in Medicine

Additional file 1: Table S2 Drug-related Anatomical Therapeutic Chemical codes

| **Drugs** | **ATC-codes** |
| --- | --- |
| Pirfenidone | L04AX05 |
| Nintedanib | L01XE31 |
| Immunosuppressants | L01XC02, L01AA01, L04AA06, L04AA10, L04A13, L04AB01, L04AB02, L04AB03, L04AB04, L04AB05, L04AB06, L04AC01, L04AC02, L04AC03, L04AC04, L04AC05, L04AC07, L04AC08, L04AC09, L04AC10, L04AC11, L04AC12, L04AX01, L04AX03 |
| Acetylcysteine | R05CB01 |
| Glucocorticoids, Corticosteroids | H02AB, R01AD |
| Sildenafil | G04BE03 |
| Treatment of pulmonary hypertension | C02KX |
| Treatment of cardiovascular disease | C10AA, C07, C09AA, C09B, C09CA, C09D |
| Treatment with anti-clotting drug | B01AC, B01AB, B01AA |
| Treatment with anti-acid drugs | A02A, A02BC, A02BA |
| Treatment with anti-depressants | N06A |
| Treatment with anti-diabetic drugs | A10A, A10B |
| Treatment with drugs against obstructive airway disease | R03AC03, R03AC12, R03AC13, R03AC18, R03AC19, R03BB01, R03BB02, R03BB03, R03BB04, R03BB05, R03BB06, R03BB07, R03BA01, R03BA02, R03BA03, R03BA04, R03BA05, R03BA06, R03BA07, R03BA08, R03BA09, R03AK06, R03AK07, R03AK08, R03AK10, R03AK11, R03AL03, R03AL04, R04AL05, R03AL06 |
| Treatment of heart insufficiency/cardiac arrhythmia | C01AA, C01BD, C03 |

*ATC: Anatomical Therapeutic Chemical*
