# Supplementary material for: Comparing outcomes of ILD patients managed in specialised versus non-specialised centres
Source: Respir Res. 2022 Aug 27;23:220. doi: 10.1186/s12931-022-02143-1 (PMC9420269; doi:10.1186/s12931-022-02143-1)
Supplement: Supplementary file 2 — Additional file 2: Table S3. Number of patients treated, patients newly treated, and procedures to classify specialised ILD centres. Table S4. Characteristics of patients with idiopathic interstitial pneumonia with raw and IPTW-weighted standardized mean differences. Table S5. Characteristics of patients with sarcoidosis with raw and IPTW-weighted standardized mean differences. Table S6. Characteristics of patients with other interstitial lung diseases with raw and IPTW-weighted standardized mean differences. Figure S1. Balancing of covariates with standardized absolute mean differences and Kolgomorov–Smirnov test. [file 12931_2022_2143_MOESM2_ESM.docx]

Additional file 2: Table S3 Number of patients treated, patients newly treated, and procedures to classify specialised ILD centres

|  | Non-specialised centres (N = 1156) | Specialised ILD centres (N = 7) |
| --- | --- | --- |
| Patients treated per year, mean (SD) | 10 (15) | 154 (26) |
| Patients newly treated per year, mean (SD) | 9 (13) | 128 (25) |
| Bronchoalveolar lavage (BAL), mean (SD) | 12 (22) | 136 (37) |
| Endobronchial ultrasound-guided transbronchial needle aspirations (EBUS-TBNA), mean (SD) | 3 (9) | 62 (16) |
| Surgical lung biopsies or transbronchial cryobiopsies, mean (SD) | 4 (11) | 116 (62) |

*SD: Standard deviation, ILD: Interstitial lung disease*

Additional file 2: Table S4 Characteristics of patients with **idiopathic interstitial pneumonia** with raw and IPTW-weighted standardized mean differences

|  | Unweighted | | | IPTW-weighted |
| --- | --- | --- | --- | --- |
|  | Non-specialised centre  (N = 11839) | Specialised  ILD centre  (N = 716) | SMD | SMD |
| Age (years), mean (SD) | 73.3 (11.5) | 69.5 (10.9) | 0.341 | <0.001 |
| Sex (female), n (%) | 4485 (37.9) | 244 (34.1) | 0.079 | <0.001 |
| GIMD 2015, n (%) |  |  | 0.601 | 0.001 |
| Q1 (least deprived quintile) | 1869 (15.8) | 246 (34.4) |  |  |
| Q2 | 2416 (20.4) | 132 (18.4) |  |  |
| Q3 | 2301 (19.4) | 33 (4.6) |  |  |
| Q4 | 2612 (22.1) | 172 (24.0) |  |  |
| Q5 (most deprived quintile) | 2641 (22.3) | 133 (18.6) |  |  |
| Residential area, n (%) |  |  | 0.404 | 0.001 |
| Major city | 2484 (21.0) | 202 (28.2) |  |  |
| Urban districts | 4304 (36.4) | 323 (45.1) |  |  |
| Rural districts | 2564 (21.7) | 132 (18.4) |  |  |
| Remote rural districts | 2487 (21.0) | 59 (8.2) |  |  |
| Nursing Home Residency, n (%) | 566 (4.8) | 15 (2.1) | 0.148 | <0.001 |
| Care dependency, n (%) |  |  | 0.348 | <0.001 |
| No care level | 8133 (68.7) | 594 (83.0) |  |  |
| Care level 1 | 1818 (15.4) | 54 (7.5) |  |  |
| Care level 2 | 1296 (10.9) | 42 (5.9) |  |  |
| Care level 3 | 445 (3.8) | 22 (3.1) |  |  |
| Care level 4 | 115 (1.0) | 3 (0.4) |  |  |
| Care level 5 | 32 (0.3) | 1 (0.1) |  |  |
| Employment, n (%) | 976 (8.2) | 109 (15.2) | 0.218 | <0.001 |
| Comorbidities Elixhauser score, mean (SD) | 3.8 (2.4) | 3.4 (2.3) | 0.180 | <0.001 |
| Comorbidities modified Elixhauser categories, n (%) |  |  |  |  |
| Congestive heart failure | 3153 (26.6) | 140 (19.6) | 0.169 | <0.001 |
| Cardiac arrhythmias | 3115 (26.3) | 137 (19.1) | 0.172 | <0.001 |
| Valvular disease | 1586 (13.4) | 70 (9.8) | 0.113 | <0.001 |
| Peripheral vascular disorders | 2818 (23.8) | 145 (20.3) | 0.086 | <0.001 |
| Hypertension, uncomplicated | 6766 (57.2) | 374 (52.2) | 0.099 | <0.001 |
| Hypertension, complicated | 1506 (12.7) | 76 (10.6) | 0.066 | <0.001 |
| Chronic pulmonary disease | 4799 (40.5) | 281 (39.2) | 0.026 | <0.001 |
| Diabetes, uncomplicated | 1773 (15.0) | 99 (13.8) | 0.033 | <0.001 |
| Diabetes, complicated | 2416 (20.4) | 151 (21.1) | 0.017 | 0.001 |
| Hypothyroidism | 1118 (9.4) | 71 (9.9) | 0.016 | <0.001 |
| Renal failure | 2293 (19.4) | 103 (14.4) | 0.133 | <0.001 |
| Liver disease | 1585 (13.4) | 111 (15.5) | 0.060 | <0.001 |
| Solid tumor without metastasis | 1353 (11.4) | 87 (12.2) | 0.022 | <0.001 |
| Rheumatoid arthritis/collagen vascular diseases | 1347 (11.4) | 72 (10.1) | 0.043 | <0.001 |
| Obesity | 1891 (16.0) | 128 (17.9) | 0.051 | <0.001 |
| Depression | 2523 (21.3) | 135 (18.9) | 0.061 | <0.001 |
| Comorbidities IPF-specific, n (%) |  |  |  |  |
| Coronary heart disease | 4227 (35.7) | 209 (29.2) | 0.139 | <0.001 |
| Gastro-oesophageal reflux disease | 1736 (14.7) | 109 (15.2) | 0.016 | <0.001 |
| Obstructive sleep apnoea syndrome | 643 (5.4) | 47 (6.6) | 0.048 | <0.001 |
| Thrombosis | 372 (3.1) | 28 (3.9) | 0.042 | <0.001 |
| Lung cancer | 342 (2.9) | 33 (4.6) | 0.091 | <0.001 |
| Pulmonary hypertension | 406 (3.4) | 25 (3.5) | 0.003 | 0.001 |
| Drug treatments, n (%) |  |  |  |  |
| Immunosuppressants | 185 (1.6) | 13 (1.8) | 0.020 | <0.001 |
| Acetylcysteine | 402 (3.4) | 30 (4.2) | 0.042 | <0.001 |
| Glucocorticoids, Corticosteroids | 2346 (19.8) | 137 (19.1) | 0.017 | <0.001 |
| Treatment with anti-clotting drugs | 2929 (24.7) | 142 (19.8) | 0.118 | <0.001 |
| Treatment with anti-acid drugs | 4764 (40.2) | 266 (37.2) | 0.063 | <0.001 |
| Treatment with anti-depressants | 1539 (13.0) | 69 (9.6) | 0.106 | <0.001 |
| Treatment with anti-diabetic drugs | 2239 (18.9) | 134 (18.7) | 0.005 | <0.001 |
| Treatment with drugs against obstructive airway disease | 2813 (23.8) | 183 (25.6) | 0.042 | 0.001 |
| Treatment of pulmonary hypertension | 58 (0.5) | 8 (1.1) | 0.070 | <0.001 |
| Treatment of heart insufficiency/cardiac arrhythmia | 4595 (38.8) | 185 (25.8) | 0.280 | <0.001 |
| Treatment of cardiovascular disease | 7542 (63.7) | 400 (55.9) | 0.160 | <0.001 |
| Hospitalisations in 3 months before treatment, n (%) |  |  |  |  |
| All cause | 4025 (34.0) | 284 (39.7) | 0.118 | 0.001 |
| Respiratory-related | 1398 (11.8) | 106 (14.8) | 0.088 | 0.001 |
| Use of outpatient services in the year before treatment |  |  |  |  |
| Contacts to physicians overall, mean (SD) | 11.1 (8.5) | 13.3 (9.0) | 0.258 | <0.001 |
| Contact to pulmonologists, n (%) | 2253 (19.0) | 314 (43.9) | 0.555 | 0.001 |
| Costs in the year before diagnosis in €, mean (SD) |  |  |  |  |
| Outpatient costs | 12745 (2392) | 1361 (2011) | 0.039 | <0.001 |
| Inpatient costs | 10106 (14332) | 11062 (20803) | 0.054 | <0.001 |
| Pharmaceutical costs | 2516 (7505) | 2463 (6446) | 0.008 | <0.001 |
| Year of confirmed diagnosis, mean (SD) |  |  | 0.139 | 0.001 |
| 2014 | 3103 (26.2) | 191 (26.7) |  |  |
| 2015 | 3000 (25.3) | 152 (21.2) |  |  |
| 2016 | 2975 (25.1) | 168 (23.5) |  |  |
| 2017 | 2761 (23.3) | 205 (28.6) |  |  |

*GIMD 2015: German Index of Multiple Deprivation, year 2015; Q: Quintile; SD: Standard deviation; SMD: Standardized mean difference*

Additional file 2: Table S5 Characteristics of patients with **sarcoidosis** with raw and IPTW-weighted standardized mean differences

|  | Unweighted | | | IPTW-weighted |
| --- | --- | --- | --- | --- |
|  | Non-specialised centre  (N = 6049) | Specialised  ILD centre  (N = 448) | SMD | SMD |
| Age (years), mean (SD) | 55.9 (16.2) | 52.7 (13.8) | 0.215 | <0.001 |
| Sex (female), n (%) | 2908 (48.1) | 203 (45.3) | 0.055 | <0.001 |
| GIMD 2015, n (%) |  |  | 0.687 | 0.001 |
| Q1 (least deprived quintile) | 965 (16.0) | 148 (33.0) |  |  |
| Q2 | 1329 (22.0) | 63 (14.1) |  |  |
| Q3 | 1234 (20.4) | 14 (3.1) |  |  |
| Q4 | 1292 (21.4) | 114 (25.4) |  |  |
| Q5 (most deprived quintile) | 1229 (20.3) | 109 (24.3) |  |  |
| Residential area, n (%) |  |  | 0.332 | 0.001 |
| Major city | 1460 (24.1) | 119 (26.6) |  |  |
| Urban districts | 2186 (36.1) | 165 (36.8) |  |  |
| Rural districts | 1213 (20.1) | 124 (27.7) |  |  |
| Remote rural districts | 1190 (19.7) | 40 (8.9) |  |  |
| Nursing Home Residency, n (%) | 64 (1.1) | 4 (0.9) | 0.017 | <0.001 |
| Care dependency, n (%) |  |  | 0.157 | <0.001 |
| No care level | 5461 (90.3) | 421 (94.0) |  |  |
| Care level 1 | 292 (4.8) | 12 (2.7) |  |  |
| Care level 2 | 212 (3.5) | 9 (2.0) |  |  |
| Care level 3 | 56 (0.9) | 5 (1.1) |  |  |
| Care level 4 | 24 (0.4) | 1 (0.2) |  |  |
| Care level 5 | 4 (0.1) | 0 (0.0) |  |  |
| Employment, n (%) | 2424 (40.1) | 243 (54.2) | 0.287 | <0.001 |
| Comorbidities Elixhauser score, mean (SD) | 2.4 (2.3) | 2.1 (2.1) | 0.139 | <0.001 |
| Comorbidities modified Elixhauser categories, n (%) |  |  |  |  |
| Congestive heart failure | 631 (10.4) | 28 (6.2) | 0.152 | <0.001 |
| Cardiac arrhythmias | 750 (12.4) | 43 (9.6) | 0.090 | <0.001 |
| Valvular disease | 380 (6.3) | 27 (6.0) | 0.011 | <0.001 |
| Peripheral vascular disorders | 587 (9.7) | 32 (7.1) | 0.092 | <0.001 |
| Hypertension, uncomplicated | 2512 (41.5) | 174 (38.8) | 0.055 | <0.001 |
| Hypertension, complicated | 430 (7.1) | 24 (5.4) | 0.072 | <0.001 |
| Chronic pulmonary disease | 1416 (23.4) | 113 (25.2) | 0.042 | <0.001 |
| Diabetes, uncomplicated | 680 (11.2) | 38 (8.5) | 0.093 | <0.001 |
| Diabetes, complicated | 823 (13.6) | 51 (11.4) | 0.067 | <0.001 |
| Hypothyroidism | 631 (10.4) | 50 (11.2) | 0.024 | <0.001 |
| Renal failure | 586 (9.7) | 21 (4.7) | 0.195 | <0.001 |
| Liver disease | 680 (11.2) | 48 (10.7) | 0.017 | <0.001 |
| Solid tumor without metastasis | 387 (6.4) | 23 (5.1) | 0.054 | 0.001 |
| Rheumatoid arthritis/collagen vascular diseases | 370 (6.1) | 24 (5.4) | 0.033 | <0.001 |
| Obesity | 1173 (19.4) | 62 (13.8) | 0.150 | <0.001 |
| Depression | 1244 (20.6) | 103 (23.0) | 0.059 | <0.001 |
| Comorbidities IPF-specific, n (%) |  |  |  |  |
| Coronary heart disease | 861 (14.2) | 43 (9.6) | 0.143 | 0.001 |
| Gastro-oesophageal reflux disease | 686 (11.3) | 45 (10.0) | 0.042 | <0.001 |
| Obstructive sleep apnoea syndrome | 274 (4.5) | 22 (4.9) | 0.018 | <0.001 |
| Thrombosis | 137 (2.3) | 11 (2.5) | 0.013 | <0.001 |
| Lung cancer | 52 (0.9) | 5 (1.1) | 0.026 | 0.001 |
| Pulmonary hypertension | 69 (1.1) | 3 (0.7) | 0.050 | 0.001 |
| Drug treatments, n (%) |  |  |  |  |
| Immunosuppressants | 75 (1.2) | 5 (1.1) | 0.011 | <0.001 |
| Acetylcysteine | 80 (1.3) | 6 (1.3) | 0.001 | <0.001 |
| Glucocorticoids, Corticosteroids | 1019 (16.8) | 56 (12.5) | 0.123 | <0.001 |
| Treatment with anti-clotting drugs | 744 (12.3) | 36 (8.0) | 0.141 | <0.001 |
| Treatment with anti-acid drugs | 1881 (31.1) | 121 (27.0) | 0.090 | <0.001 |
| Treatment with anti-depressants | 691 (11.4) | 39 (8.7) | 0.090 | <0.001 |
| Treatment with anti-diabetic drugs | 927 (15.3) | 47 (10.5) | 0.145 | <0.001 |
| Treatment with drugs against obstructive airway disease | 890 (14.7) | 74 (16.5) | 0.050 | 0.001 |
| Treatment of pulmonary hypertension | 5 (0.1) | 1 (0.2) | 0.036 | <0.001 |
| Treatment of heart insufficiency/cardiac arrhythmia | 1077 (17.8) | 49 (10.9) | 0.197 | 0.001 |
| Treatment of cardiovascular disease | 2561 (42.3) | 167 (37.3) | 0.104 | <0.001 |
| Hospitalisations in 3 months before treatment, n (%) |  |  |  |  |
| All cause | 1937 (32.0) | 144 (32.1) | 0.003 | 0.001 |
| Respiratory-related | 264 (4.4) | 17 (3.8) | 0.029 | <0.001 |
| Use of outpatient services in the year before treatment |  |  |  |  |
| Contacts to physicians overall, mean (SD) | 11.6 (8.0) | 12.6 (7.2) | 0.132 | <0.001 |
| Contact to pulmonologists, n (%) | 1184 (19.6) | 157 (35.0) | 0.353 | <0.001 |
| Costs in the year before diagnosis in €, mean (SD) |  |  |  |  |
| Outpatient costs | 1095 (2248) | 1019 (1383) | 0.041 | 0.001 |
| Inpatient costs | 6966 (8892) | 6865 (10350) | 0.010 | <0.001 |
| Pharmaceutical costs | 1423 (5742) | 1330 (5523) | 0.017 | <0.001 |
| Year of confirmed diagnosis, mean (SD) |  |  | 0.094 | <0.001 |
| 2014 | 1528 (25.3) | 101 (22.5) |  |  |
| 2015 | 1536 (25.4) | 115 (25.7) |  |  |
| 2016 | 1517 (25.1) | 107 (23.9) |  |  |
| 2017 | 1468 (24.3) | 125 (27.9) |  |  |

*GIMD 2015: German Index of Multiple Deprivation, year 2015; Q: Quintile; SD: Standard deviation; SMD: Standardized mean difference*

Additional file 2: Table S6 Characteristics of patients with **other interstitial lung diseases** with raw and IPTW-weighted standardized mean differences

|  | Unweighted | | | IPTW-weighted |
| --- | --- | --- | --- | --- |
|  | Non-specialised centre  (N = 10883) | Specialised  ILD centre  (N =858) | SMD | SMD |
| Age (years), mean (SD) | 67.3 (14.0) | 65.3 (13.3) | 0.144 | <0.001 |
| Sex (female), n (%) | 4913 (45.1) | 332 (38.7) | 0.131 | <0.001 |
| ILD entity [ICD-10], n (%) |  |  | 0.331 | 0.001 |
| Other fibrosing ILDs [J84.0, J84.8, J84.9, D48.1] | 6147 (56.5) | 507 (59.1) |  |  |
| Drug-associated ILDs [J70.2-J70.4] | 488 (4.5) | 37 (4.3) |  |  |
| Pneumoconiosis [J62.0-J62.8, J63.0-J63.8] | 826 (7.6) | 38 (4.4) |  |  |
| Radiation-associated pneumonitis [J70.1] | 438 (4.0) | 63 (7.3) |  |  |
| Eosinophilic pneumonia [J82] | 624 (5.7) | 54 (6.3) |  |  |
| Hypersensitivity pneumonitis [J67.9] | 773 (7.1) | 97 (11.3) |  |  |
| Connective tissue-associated ILD [J99.1] | 1587 (14.6) | 62 (7.2) |  |  |
| GIMD 2015, n (%) |  |  | 0.589 | 0.001 |
| Q1 (least deprived quintile) | 1822 (16.7) | 256 (29.8) |  |  |
| Q2 | 2386 (21.9) | 141 (16.4) |  |  |
| Q3 | 2143 (19.7) | 33 (3.8) |  |  |
| Q4 | 2217 (20.4) | 221 (25.8) |  |  |
| Q5 (most deprived quintile) | 2315 (21.3) | 207 (24.1) |  |  |
| Residential area, n (%) |  |  | 0.274 | 0.001 |
| Major city | 2572 (23.6) | 241 (28.1) |  |  |
| Urban districts | 3995 (36.7) | 298 (34.7) |  |  |
| Rural districts | 2246 (20.6) | 229 (26.7) |  |  |
| Remote rural districts | 2070 (19.0) | 90 (10.5) |  |  |
| Nursing Home Residency, n (%) | 345 (3.2) | 6 (0.7) | 0.180 | 0.002 |
| Care dependency, n (%) |  |  | 0.260 | 0.001 |
| No care level | 8360 (76.8) | 743 (86.6) |  |  |
| Care level 1 | 1143 (10.5) | 46 (5.4) |  |  |
| Care level 2 | 941 (8.6) | 47 (5.5) |  |  |
| Care level 3 | 322 (3.0) | 15 (1.7) |  |  |
| Care level 4 | 93 (0.9) | 5 (0.6) |  |  |
| Care level 5 | 24 (0.2) | 2 (0.2) |  |  |
| Employment, n (%) | 1756 (16.1) | 175 (20.4) | 0.110 | <0.001 |
| Comorbidities Elixhauser score, mean (SD) | 3.5 (2.4) | 3.3 (2.3) | 0.108 | <0.001 |
| Comorbidities modified Elixhauser categories, n (%) |  |  |  |  |
| Congestive heart failure | 2136 (19.6) | 137 (16.0) | 0.096 | <0.001 |
| Cardiac arrhythmias | 2413 (22.2) | 154 (17.9) | 0.106 | 0.001 |
| Valvular disease | 1241 (11.4) | 89 (10.4) | 0.033 | <0.001 |
| Peripheral vascular disorders | 1996 (18.3) | 132 (15.4) | 0.079 | 0.001 |
| Hypertension, uncomplicated | 5603 (51.5) | 420 (49.0) | 0.051 | <0.001 |
| Hypertension, complicated | 1202 (11.0) | 100 (11.7) | 0.019 | 0.001 |
| Chronic pulmonary disease | 4046 (37.2) | 351 (40.9) | 0.077 | 0.001 |
| Diabetes, uncomplicated | 1396 (12.8) | 92 (10.7) | 0.065 | <0.001 |
| Diabetes, complicated | 1852 (17.0) | 121 (14.1) | 0.080 | <0.001 |
| Hypothyroidism | 1207 (11.1) | 97 (11.3) | 0.007 | <0.001 |
| Renal failure | 1762 (16.2) | 117 (13.6) | 0.072 | <0.001 |
| Liver disease | 1431 (13.1) | 125 (14.6) | 0.041 | <0.001 |
| Solid tumor without metastasis | 1259 (11.6) | 89 (10.4) | 0.038 | 0.001 |
| Rheumatoid arthritis/collagen vascular diseases | 1887 (17.3) | 105 (12.2) | 0.144 | <0.001 |
| Obesity | 1836 (16.9) | 134 (15.6) | 0.034 | <0.001 |
| Depression | 2423 (22.3) | 198 (23.1) | 0.019 | <0.001 |
| Comorbidities IPF-specific, n (%) |  |  |  |  |
| Coronary heart disease | 2797 (25.7) | 206 (24.0) | 0.039 | <0.001 |
| Gastro-oesophageal reflux disease | 1517 (13.9) | 139 (16.2) | 0.063 | <0.001 |
| Obstructive sleep apnoea syndrome | 610 (5.6) | 71 (8.3) | 0.105 | <0.001 |
| Thrombosis | 343 (3.2) | 29 (3.4) | 0.013 | <0.001 |
| Lung cancer | 622 (5.7) | 108 (12.6) | 0.240 | 0.001 |
| Pulmonary hypertension | 369 (3.4) | 36 (4.2) | 0.042 | <0.001 |
| Drug treatments, n (%) |  |  |  |  |
| Immunosuppressants | 412 (3.8) | 21 (2.4) | 0.077 | <0.001 |
| Acetylcysteine | 321 (2.9) | 43 (5.0) | 0.106 | 0.001 |
| Glucocorticoids, Corticosteroids | 2742 (25.2) | 224 (26.1) | 0.021 | <0.001 |
| Treatment with anti-clotting drugs | 2177 (20.0) | 144 (16.8) | 0.083 | <0.001 |
| Treatment with anti-acid drugs | 4456 (40.9) | 308 (35.9) | 0.104 | <0.001 |
| Treatment with anti-depressants | 1390 (12.8) | 84 (9.8) | 0.094 | <0.001 |
| Treatment with anti-diabetic drugs | 1711 (15.7) | 117 (13.6) | 0.059 | <0.001 |
| Treatment with drugs against obstructive airway disease | 2401 (22.1) | 217 (25.3) | 0.076 | 0.001 |
| Treatment of pulmonary hypertension | 128 (1.2) | 6 (0.7) | 0.049 | <0.001 |
| Treatment of heart insufficiency/cardiac arrhythmia | 3347 (30.8) | 215 (25.1) | 0.127 | <0.001 |
| Treatment of cardiovascular disease | 6115 (56.2) | 428 (49.9) | 0.127 | 0.001 |
| Hospitalisations in three months before treatment, n (%) |  |  |  |  |
| All cause | 4370 (40.2) | 419 (48.8) | 0.175 | <0.001 |
| Respiratory-related | 1377 (12.7) | 137 (16.0) | 0.095 | <0.001 |
| Use of outpatient services in the year before treatment |  |  |  |  |
| Contacts to physicians overall, mean (SD) | 12.7 (9.8) | 12.4 (7.9) | 0.026 | <0.001 |
| Contact to pulmonologists, n (%) | 2236 (20.5) | 359 (41.8) | 0.472 | 0.002 |
| Costs in the year before diagnosis in €, mean (SD) |  |  |  |  |
| Outpatient costs | 1547 (2971) | 1367 (1926) | 0.072 | 0.001 |
| Inpatient costs | 11782 (17681) | 11074 (17294) | 0.041 | <0.001 |
| Pharmaceutical costs | 3462 (10198) | 2706 (8251) | 0.082 | <0.001 |
| Year of confirmed diagnosis, mean (SD) |  |  | 0.163 | <0.001 |
| 2014 | 2702 (24.8) | 175 (20.4) |  |  |
| 2015 | 2646 (24.3) | 190 (22.1) |  |  |
| 2016 | 2745 (25.2) | 215 (25.1) |  |  |
| 2017 | 2790 (25.6) | 278 (32.4) |  |  |

*GIMD 2015: German Index of Multiple Deprivation, year 2015; Q: Quintile; SD: Standard deviation; SMD: Standardized mean difference*


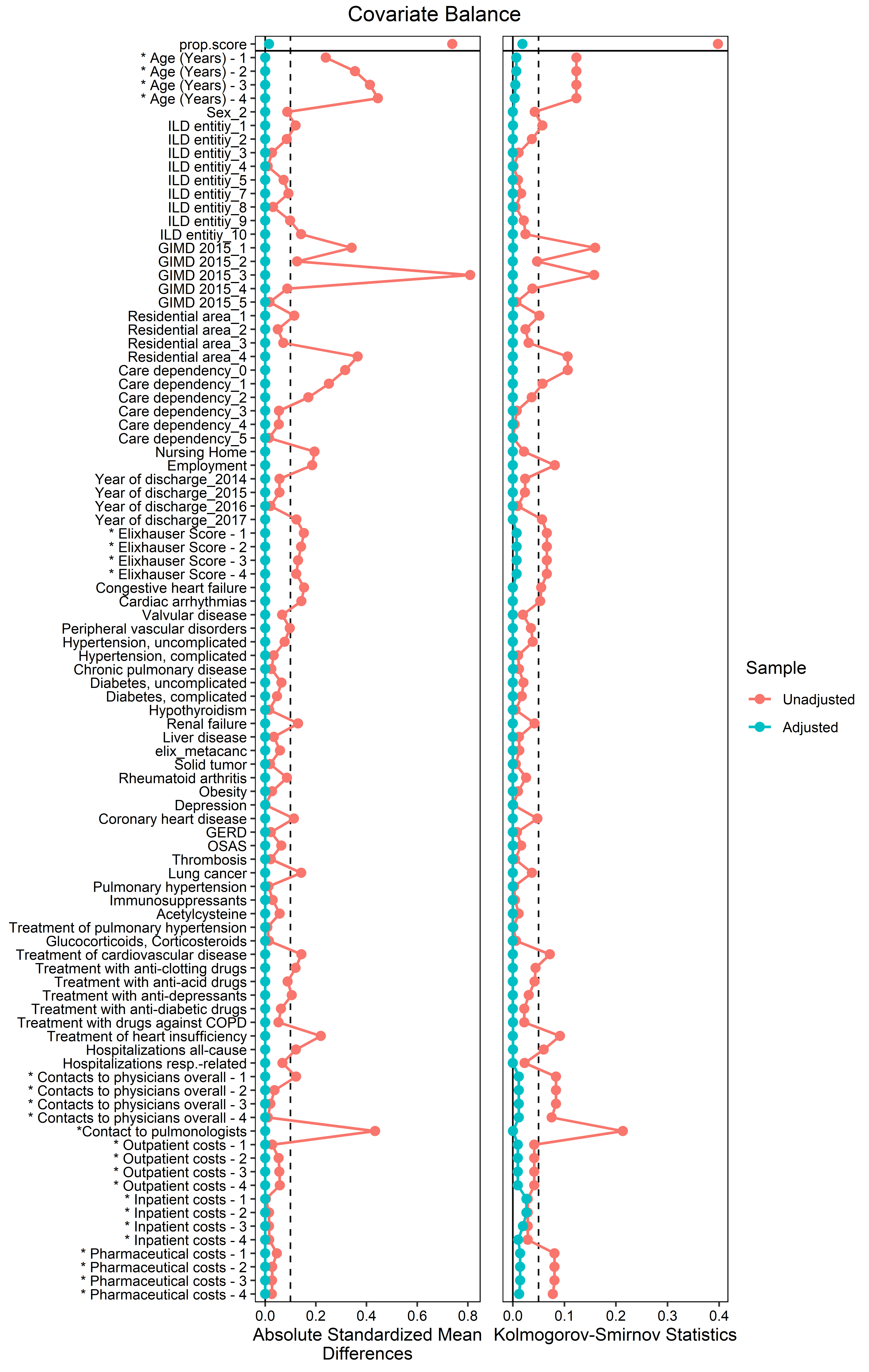


Additional file 2: Figure S1 Balancing of covariates with standardized absolute mean differences and Kolgomorov-Smirnov test

*Standardized Mean Difference: Threshold at 0.1. Higher values indicate imbalance.*

*Kolgomorov-Smirnov test for continuous variables: Threshold at 0.05. Higher values indicate imbalance.*

** Continuous variables*
