# Supplementary material for: Comparing outcomes of ILD patients managed in specialised versus non-specialised centres
Source: Respir Res. 2022 Aug 27;23:220. doi: 10.1186/s12931-022-02143-1 (PMC9420269; doi:10.1186/s12931-022-02143-1)
Supplement: Supplementary file 3 — Additional file 3: Table S7. Estimates of unweighted Cox Proportional Hazard Models for 2-year mortality and 1-year hospitalisation after confirmed diagnosis for main analysis and subgroups. Table S8. IPTW-weighted model estimated expenditures of 1-year costs and related cost differences for patients with idiopathic interstitial pneumonia. Table S9. IPTW-weighted model estimated expenditures of 1-year costs and related cost differences for patients with sarcoidosis. Table S10. IPTW-weighted model estimated expenditures of 1-year costs and related cost differences for patients with other interstitial lung diseases. [file 12931_2022_2143_MOESM3_ESM.docx]

Additional file 3: Table S7 Estimates of unweighted Cox Proportional Hazard Models for two-year mortality and one-year hospitalisation after confirmed diagnosis for main analysis and subgroups

|  |  | | *Subgroup Analyses* | | | | |
| --- | --- | --- | --- | --- | --- | --- | --- |
|  | *All patients* | | *Idiopathic Interstitial Pneumonia* | | *Sarcoidosis* | | *Other interstitial lung diseases* |
|  | *HR (95%-CI)* | | *HR (95%-CI)* | | *HR (95%-CI)* | | *HR (95%-CI)* |
| **2-year all-cause survival** |  |  |  |  |  |  |  |
| Specialised ILD-centre vs. non-specialised centre | 0.69 (0.62, 0.76)* | | 0.64 (0.55, 0.74)* | | 0.58 (0.39, 0.87)* | | 0.79 (0.69, 0.92)* |
| **1-year all-cause hospitalisation** |  |  |  |  |  |  |  |
| Specialised ILD-centre vs. non-specialised centre | 0.83 (0.79, 0.88)* | | 0.82 (0.74, 0.90)* | | 0.79 (0.68, 0.92)* | | 0.86 (0.79, 0.94)* |
| **1-year respiratory-related hospitalisation** |  |  |  |  |  |  |  |
| Specialised ILD-centre vs. non-specialised centre | 0.90 (0.82, 0.98)* | | 0.89 (0.78, 1.01) | | 0.75 (0.59, 0.95)* | | 1.02 (0.89, 1.17) |

*CI: confidence interval, HR: Hazard Ratio*

** Statistically significant results*

Additional file 3: Table S8 IPTW-weighted model estimated expenditures of one-year costs and related cost differences for patients with **idiopathic interstitial pneumonia**

|  | Non-specialised centre | Specialised ILD-centre |  |
| --- | --- | --- | --- |
|  | Costs in € (95% CI) | Costs in € (95% CI) | *Difference (in €)* |
| **Overall** |  |  |  |
| Total | 16,478 (15,708; 17,402) | 16,667 (15,184; 18,267) | 189 (-1,525; 1,884) |
| Inpatient | 9,829 (9,188; 10,615) | 8,059 (6,869; 9,401) | -1,770 (-3,131; -343)* |
| Outpatient | 1,425 (1,364; 1,488) | 1,318 (1,219; 1,446) | -106 (-225; 27) |
| Pharmaceuticals | 5,225 (4,813; 5,717) | 7,282 (6,396; 8,181) | 2,058 (1,083; 3,106)* |
|  |  |  |  |
| **Respiratory-related** |  |  |  |
| Total | 6,381 (5,799; 7,110) | 7,695 (6,809; 8,711) | 1314 (177; 2,500)* |
| Inpatient | 3,311 ( 2,895; 3,911) | 2,336 (1,906; 2,842) | -975 (-1,708; -302)* |
| Outpatient | 550 (513; 592) | 502 (474; 531) | -47 (-96; 2) |
| Pharmaceuticals | 2,520 (2,196; 2,907) | 4,856 (4,093; 5,654) | 2,336 (1,493; 3,209)* |

*CI: Confidence interval, ILD: interstitial lung disease*

*Estimation based on weighted one and two-part generalized linear gamma models via recycled predictions approach with 1000 bootstrap replicates*

** Statistically significant results*

Additional file 3: Table S9 IPTW-weighted model estimated expenditures of one-year costs and related cost differences for patients with **sarcoidosis**

|  | Non-specialised centre | Specialised ILD-centre |  |
| --- | --- | --- | --- |
|  | Costs in € (95% CI) | Costs in € (95% CI) | *Difference (in €)* |
| **Overall** |  |  |  |
| Total | 7,478 (6,999; 7,869) | 5,836 (5,165; 6,989) | -1,642 (-2,398; -10)* |
| Inpatient | 4,648 (4,195; 5,151) | 3,119 (2,471; 3,872) | -1,529 (-2,383; -641)* |
| Outpatient | 1,218 (1,177; 1,263) | 1,177 (1,057; 1,327) | -42 (-173; 116) |
| Pharmaceuticals | 1,861 (1,652; 2,127) | 1,841 (1,289; 2,458) | -19 (-618; 663) |
|  |  |  |  |
| **Respiratory-related** |  |  |  |
| Total | 1,755 (1,608; 1,915) | 1,544 (1,208; 1,978) | -211 (-571; 228) |
| Inpatient | 1,059 ( 927; 1,195) | 849 (588; 1,149) | -210 (-481; 117) |
| Outpatient | 519 (499; 541) | 499 (470; 530) | -20 (-57; 17) |
| Pharmaceuticals | 177 (120; 251) | 195 (36; 525) | 19 (-176; 342) |

*CI: Confidence interval, ILD: interstitial lung disease*

*Estimation based on weighted one and two-part generalized linear gamma models via recycled predictions approach with 1000 bootstrap replicates*

** Statistically significant results*

Additional file 3: Table S10 IPTW-weighted model estimated expenditures of one-year costs and related cost differences for patients with **other interstitial lung diseases**

|  | Non-specialised centre | Specialised ILD-centre |  |
| --- | --- | --- | --- |
|  | Costs in € (95% CI) | Costs in € (95% CI) | *Difference (in €)* |
| **Overall** |  |  |  |
| Total | 15,555 (14,892; 16,207) | 13,732 (12,477; 15,042) | -1,823 (-3,220; -369)* |
| Inpatient | 10,245 (9,721; 10,772) | 8,069 (7,215; 9,024) | -2,176 (-3,260; -1,123)* |
| Outpatient | 1,436 (1,389; 1,486) | 1,491 (1,314; 1,706) | 55 (-136; 267) |
| Pharmaceuticals | 3,874 (3,547; 4,236) | 4,171 (3,458; 4,976) | 297 (-508; 1,138) |
|  |  |  |  |
| **Respiratory-related** |  |  |  |
| Total | 2,943 (2,770; 3,137) | 3,100 (2,561; 3,707) | 157 (-409; 783) |
| Inpatient | 2,046 ( 1,881; 2,229) | 1,764 (1,442; 2,148) | -282 (-660; 150) |
| Outpatient | 422 (402; 445) | 493 (390; 633) | 71 (-32; 213) |
| Pharmaceuticals | 475 (411; 542) | 843 (486; 1,261) | 369 (-7; 790) |

*CI: Confidence interval, ILD: interstitial lung disease*

*Estimation based on weighted one and two-part generalized linear gamma models via recycled predictions approach with 1000 bootstrap replicates*

** Statistically significant results*
