# Supplementary material for: Comparing outcomes of ILD patients managed in specialised versus non-specialised centres
Source: Respir Res. 2022 Aug 27;23:220. doi: 10.1186/s12931-022-02143-1 (PMC9420269; doi:10.1186/s12931-022-02143-1)
Supplement: Supplementary file 4 — Additional file 4: Table S11. Explorative analysis for change in pharmaceutical treatment patterns in the 3 month after confirmed diagnosis. [file 12931_2022_2143_MOESM4_ESM.docx]

Additional file 4: Table S11 Explorative analysis for change in pharmaceutical treatment patterns in the three month after confirmed diagnosis

|  | Percentage treated with drugs before confirmed diagnosis, n (%) | | Percentage treated with drugs after confirmed discharge, n (%) | |
| --- | --- | --- | --- | --- |
|  | Non-specialised ILD-centre | Specialised ILD-centre | Non-specialised ILD-centre | Specialised ILD-centre |
| Antifibrotics | 0 (0) | 0 (0) | 350 (1.2) | 84 (4.2) |
| Immunosuppressants | 672 (2.3) | 39 (1.9) | 963 (3.3) | 76 (3.8) |
| Acetylcysteine | 803 (2.8) | 79 (3.9) | 1102 (3.8) | 91 (4.5) |
| Glucocorticoids, Corticosteroids | 6107 (21.2) | 417 (20.6) | 12173 (42.3) | 896 (44.3) |
| Treatment with anti-clotting drugs | 5850 (20.3) | 322 (15.9) | 6962 (24.2) | 357 (17.7) |
| Treatment with anti-acid drugs | 11101 (38.6) | 695 (34.4) | 15188 (52.8) | 981 (48.5) |
| Treatment with anti-depressants | 3620 (12.6) | 192 (9.5) | 4033 (14.0) | 212 (10.5) |
| Treatment with anti-diabetic drugs | 4877 (17.0) | 298 (14.7) | 5092 (17.7) | 320 (15.8) |
| Treatment with drugs against obstructive airway disease | 6104 (21.2) | 474 (23.4) | 7024 (24.4) | 418 (20.7) |
| Treatment of pulmonary hypertension | 191 (0.7) | 15 (0.7) | 350 (1.2) | 40 (2.0) |
| Treatment of heart insufficiency/cardiac arrhythmia | 9019 (31.3) | 449 (22.2) | 11200 (38.9) | 519 (25.7) |
| Treatment of cardiovascular disease | 16218 (56.4) | 995 (49.2) | 16746 (58.2) | 1030 (50.9) |
|  |  |  |  |  |

*ILD: Interstitial lung disease*
