# Supplementary material for: Comparing outcomes of ILD patients managed in specialised versus non-specialised centres
Source: Respir Res. 2022 Aug 27;23:220. doi: 10.1186/s12931-022-02143-1 (PMC9420269; doi:10.1186/s12931-022-02143-1)
Supplement: Supplementary file 5 — Additional file 5: Table S12. Sensitivity analyses with unweighted and IPTW-weighted Cox Proportional Hazard models for 2-year mortality and 1-year all-cause and respiratory-related hospitalisation. [file 12931_2022_2143_MOESM5_ESM.docx]

Additional file 5: Table S12 Sensitivity analyses with unweighted and IPTW-weighted Cox Proportional Hazard models for 2-year mortality and 1-year all-cause and respiratory-related hospitalisation

|  | *Unweighted* | | *IPTW-Weighted* | |
| --- | --- | --- | --- | --- |
|  | *HR*  *(95%-CI)* | | *HR*  *(95%-CI)* | |
| **2-year all-cause survival** |  |  |  |  |
| Specialised ILD-centre vs. non-specialised centre | 0.69 (0.62, 0.76)* | | 0.88 (0.79, 0.97)* | |
| **1-year all-cause hospitalisation** |  |  |  |  |
| Specialised ILD-centre vs. non-specialised centre | 0.83 (0.79, 0.88)* | | 0.93 (0.87, 0.98)* | |
| **1-year respiratory-related hospitalisation** |  |  |  |  |
| Specialised ILD-centre vs. non-specialised centre | 0.90 (0.82, 0.98)* | | 1.01 (0.92, 1.10) | |

*CI: Confidence interval, HR: Hazard Ratio, ILD: Interstitial lung disease*
